# The Effect of Telemedicine on Access to Acute Stroke Care in Texas: The Story of Age Inequalities

**DOI:** 10.1155/2015/813493

**Published:** 2015-10-12

**Authors:** Karen C. Albright, Amelia K. Boehme, Michael T. Mullen, Tzu-Ching Wu, Charles C. Branas, James C. Grotta, Sean I. Savitz, Catherine Wolff, Bisakha Sen, Brendan G. Carr

**Affiliations:** ^1^Geriatric Research, Education and Clinical Center (GRECC), Birmingham VA Medical Center, Birmingham, AL 35249, USA; ^2^Department of Epidemiology, School of Public Health, University of Alabama at Birmingham, Birmingham, AL 35249, USA; ^3^Department of Neurology, Columbia University, New York, NY 10032, USA; ^4^Department of Neurology, University of Pennsylvania, Philadelphia, PA 19104, USA; ^5^Stroke Program, Department of Neurology, University of Texas-Houston Memorial Hermann Medical Center, Houston, TX 77030, USA; ^6^Department of Clinical Epidemiology and Biostatistics, University of Pennsylvania, Philadelphia, PA 19104, USA; ^7^Department of Health Care Organization and Policy, School of Public Health, University of Alabama at Birmingham, Birmingham, AL 35249, USA; ^8^Department of Emergency Medicine, University of Pennsylvania, Philadelphia, PA 19104, USA

## Abstract

*Background*. Ischemic stroke is a time sensitive disease with the effectiveness of treatment decreasing over time. Treatment is more likely to occur at Primary Stroke Centers (PSC); thus rapid access to acute stroke care through stand-alone PSCs or telemedicine (TM) is vital for all Americans. The objective of this study is to determine if disparities exist in access to PSCs or the extended access to acute stroke care provided by TM. *Methods*. Data from the US Census Bureau and the 2010 Neilson Claritas Demographic Estimation Program, American Hospital Association annual survey, and The Joint Commission list of PSCs and survey response data for all hospitals in the state of Texas were used. *Results*. Over 64% of block groups had 60-minute ground access to acute stroke care. The odds of a block group having 60-minute access to acute stroke care decreased with age, despite adjustment for sex, race, ethnicity, socioeconomic status, urbanization, and total population. *Conclusion*. Our survey of Texas hospitals found that as the median age of a block group increased, the odds of having access to acute stroke care decreased.

## 1. Introduction

Stroke is the leading cause of adult disability in the United States [1]. Approximately 800,000 people have a stroke each year, with total direct and indirect costs exceeding $38 billion [2]. The burden of stroke is expected to increase as the population ages, and, by 2030, it is projected that stroke prevalence will increase by nearly 22% [2]. The elderly who reside in rural areas, particularly if the rural area consists of lower socioeconomic status groups, have worse access to care [3]. Despite the provision of Medicare, there are still inequalities associated with access to care for the elderly [4, 5].

Timely access to acute stroke care is vital for effective stroke treatment given that the only FDA approved treatment must be administered within 4.5 hours of symptom onset [6, 7]. Hospitals certified as Primary Stroke Centers (PSCs) have been shown to administer significantly more t-PA than noncertified hospitals [8]. PSC certification can be resource intensive, particularly for small hospitals or hospitals in remote locations. Telemedicine (TM), which allows a neurologist at a distant site to interact with the patient and his/her family at an outlying hospital, has been proposed as a means of increasing access to care in these areas [9]. Telemedicine can be used to support PSC certification or to provide acute stroke care at hospitals that are not certified as PSCs. Prior studies have shown that the use of telemedicine for treatment of ischemic stroke not only is safe and effective but also increases the utilization of t-PA, thereby improving patient care and outcomes [10–12].

Given the burden caused by stroke and the aging of our population, improving stroke care is a public health priority [13, 14]. While studies have investigated access to primary care among the elderly, no study has evaluated their access to acute stroke care [3]. Furthermore, it is unclear whether telemedicine networks are developing to effectively target at risk populations. We hypothesized that disparities in access to acute stroke care exist for the elderly. The objectives of the study were to determine if differences exist in access to acute stroke care for the elderly and to determine the role that telemedicine had in these differences.

## 2. Methods

### 2.1. Study Population

The details of the survey methodology have been previously described [15]. Population demographic information for 2010 was obtained from the US Census Bureau and from Neilson Claritas [16]. Age (median and categorical), sex, race/ethnicity, median household income, proportion with less than a high school education, proportion under 65 years of age without health insurance, and urbanization were examined. Urbanization was defined according to a modification of the rural-urban continuum classification (mRUC) scheme [17–20]. The mRUC scheme is a county level variable scale of 1–9 that defines urbanization. The primary unit of analysis was the block group, a small unit of geographic analysis that is contained within the boundaries of a census tract and comprised of a population of 600–3000 people. Calculations were conducted from the block group centroid, which is the population weighed center.

### 2.2. Access to Acute Stroke Care

As previously reported, Texas hospitals and resources were identified from the 2009 American Hospital Association Annual (AHA) Survey [15]. Hospitals with emergency departments that manage adult patients were defined as acute care facilities. Access to acute stroke care was defined as having a stand-alone primary stroke center (PSC) or having access to stroke specialists via telemedicine. PSC status was determined by identifying state certified hospitals and hospitals certified by The Joint Commission (TJC) from the list of acute care facilities [21]. Telemedicine (TM) use was determined by phone interview (detailed methodology previously described) [15]. Using a standardized questionnaire, interviewees were asked if their hospital was (1) an acute care facility, (2) a PSC, and (3) currently using TM and (4) whether TM was being used for acute stroke management. Self-reported TJC PSC status was cross-checked with a publicly available listing of TJC accredited PSCs and state designated comprehensive stroke centers [22–24]. Once validated, hospitals were categorized as a stand-alone PSC, a PSC requiring TM for acute stroke care, or a TM capable hospital not utilizing TM for acute stroke care. Each block group was categorized as having “duplicate access” (access to PSC and acute stroke care via TM), “single access” (access to either a PSC or acute stroke care via TM), “potential access” (access to a hospital where TM was in use for something other than acute stroke), or “no access” to acute stroke care.

### 2.3. Access to Care Calculations

In this ecologic analysis, access to care was defined as block groups that were within 60 minutes of acute stroke care [25]. Time estimates for each block group were calculated using the distance from the block group centroid to the closest hospital of each type, with the addition of validated ambulance prehospital time intervals [25]. The population of block groups within these catchments was summed to determine total population access within 60 minutes. Calculations were restricted to only consider the population and facilities residing within Texas state borders.

The Network Analyst functionality in ESRI ArcMap 10.1 was used to determine the shortest road distance between each centroid and the nearest hospital [26, 27]. Transport times were calculated based on posted speed limits with 10 mph added to the speed limit for the roads in each path to the linked hospital. Key prehospital ambulance time intervals, adjusted based on urbanization, were added to the transport time to estimate the total prehospital travel time. The 911 activation to ambulance dispatch interval was estimated as 1.4, 1.4, and 2.9 minutes for urban, suburban, and rural areas, respectively [25]. The time from ambulance dispatch until arrival at the scene was determined by multiplying the drive time from the scene to the hospital (as described above) by 1.6, 1.5, and 1.4 for urban, suburban, and rural drives, respectively [25]. Lastly, 13.5, 13.5, and 15.1 minutes were added to account for time spent by emergency medical services (EMS) on the scene prior to transport for urban, suburban, and rural areas as has been done previously [22, 28].

### 2.4. Statistics

Given that the proportion with access to a stand-alone PSC was greater than 20% in all groups, we elected to use modified Poisson regression to produce prevalence odds ratios (POR) to illustrate the association between race and ethnicity and access to acute stroke care [23]. This decision was made in an attempt to prevent the overestimation often seen when using logistic regression (odds ratios) in situations with prevalent outcomes [23].

The proportion of block groups with 60-minute access to a hospital providing acute stroke care via TM that also had 60-minute access to a PSC was compared to the proportion of block groups with 60-minute access to a hospital providing acute stroke care via TM that did not have 60-minute access to a PSC using Chi-square. Four logistic regression models were created to determine if median age, sex, race/ethnicity, median household income, proportion with less than a high school education, proportion under 65 years of age without health insurance, or urbanization were predictors of having 60-minute access to acute stroke care. Model 1 examined age with no adjustment. Model 2 examined age, adjusting for race and ethnicity. Model 3 examined age, adjusting for race, ethnicity, and two socioeconomic status (SES) proxies (i.e., median household income and proportion with less than a high school education). Model 4 examined age, adjusting for race, ethnicity, both SES proxies, sex, insurance, and degree of urbanization. Multinomial logistic regression was used to assess the association between previously described predictors and categories of access (duplicate, single, and potential) as compared to no access, using the same four models.

## 3. Results

A total of 578 Texas hospitals were identified in the AHA database, of which 96% (556/578) completed the telephone survey [15]. Twenty-two hospitals did not participate (4 hospitals elected not to participate and 18 could not be reached by phone). Of the 556 hospitals completing the survey, 395 (71%) were identified as adult acute care facilities. Of the acute care facilities, 76 (19%) were confirmed as TJC-certified PSC. Twenty-two (29%) of these facilities reported using TM to deliver acute stroke care [15]. Additional 26 sites were using TM but not for acute stroke care [15].

Overall 64% of block groups (*n* = 9, 297) had 60-minute access to acute stroke care, either a stand-alone PSC or a hospital using TM for acute stroke. Telemedicine was found in 63% with stand-alone PSC access and only 25% of block groups without access to stand-alone PSCs (*p* < 0.0001). Nearly 44% of block groups (*n* = 6, 303) had “duplicate access,” and approximately 21% (*n* = 2, 994) had “single access,” while nearly 16% (*n* = 2, 268) had “potential access,” and 20% (*n* = 2, 911) had “no access.” As shown in Table 1, a larger proportion of those with more than 65 years of age had no access to acute stroke care. Median age was found to be the only significant predictor of 60-minute access to acute stroke care in all four logistic prediction models (Table 2, *p* < 0.001). For each 10-year increase in age, the odds of having 60-minute access to acute stroke care decreased by 5%.

The fully adjusted multinomial logistic regression model revealed that the odds of a block group having duplicate access compared to no access were significantly lower as age increased (Table 3, *p* < 0.001). Lower odds of duplicate access were also seen as the population increased, as the proportion with less than a high school education increased, as the proportion under age of 65 without health insurance increased, and as urbanization decreased. The odds of a block group having single access when compared to no access were significantly lower as age increased, as the population increased, as the proportion with less than a high school education increased, and as urbanization decreased (Table 3, *p* < 0.001). In the fully adjusted multinomial logistic regression model, the odds of a block group having potential access were significantly lower as the median age of the block group increased, as the population increased, as the proportion with less than a high school education increased, as the proportion under age of 65 without health insurance increased, and as urbanization decreased (Table 3, *p* < 0.001).

## 4. Discussion

Our study survey of Texas hospitals found that 64% of Texans have 60-minute ground access to acute stroke care, either via a stand-alone PSC or by using TM to connect with distant stroke experts. In the crude models, older age was associated with decreased access to acute stroke care. This disparity remained even after adjustment for race, ethnicity, gender, socioeconomic proxies, insurance coverage, urbanization, and total population.

We found that telemedicine centers and stand-alone PSCs were geographically clustered. The fact that 63% of block groups with access to a stand-alone PSC also had access to a TM site raises concern that a potential predictor of getting a TM center is having a stand-alone PSC. Furthermore, the fact that only 24% of block groups without access to stand-alone PSCs had access to TM suggests that this self-evolved system may not be targeting population needs.

We found that older adults had poor access to acute stroke care of all types. Additionally, we found that as age increases, the odds of access to a stand-alone PSC or a TM site providing acute stroke care decrease. Our findings are consistent with previous research indicating that the elderly have decreased access to care [3]. It may be more appropriate to use the number of older adults as a proxy for those at risk for stroke. The distribution of TM sites providing acute stroke care may be the result of initiatives to focus funds and resources on other disease states or the impression that there is sufficient acute stroke coverage already in an area. It is also possible that other aspects related to patterns of where individuals live during their postretirement years (but not reflected in current studies) result in a disproportionate ratio of “underserved” individuals who live outside areas of service; a better understanding of this potential phenomenon may help to guide allocation of resources. The drivers of telemedicine placement are understudied and poorly understood. Reimbursement is likely at play, however the age effect remained even after adjusting for insurance coverage.

As the aging population continues to expand, the issues of access to acute stroke care for the elderly illustrate an enormous looming public health issue. Without timely treatment, acute stroke patients are likely to experience worse outcome, resulting in increased morbidity and mortality.

Although this analysis is restricted to Texas, access to stroke care is of national importance. This study is illustrious of the potential barriers that exist in all states, whether they be densely urban, diffusely populated, or with the mixed centers of urbanization and rurality which is characteristic of Texas, although our findings may not be generalizable to the other 49 states. To further determine how public policy can move toward a goal of providing sufficient care to the greatest amount of the populace which requires a critical review of what services currently exist is necessary. Public policy interventions should be able to improve access to care by incentivizing certification, with or without TM, in areas of great need.

Our study is not without limitations. Our study determined access to stand-alone TJC-certified PSCs or state certified PSCs. It did not include hospitals participating in national quality-improvement programs [24]. Furthermore, we used transport times empirically derived from trauma. Drive times for trauma care are, on average, 6 to 11 minutes shorter than those for patients with stroke [29, 30]. Our estimates of access were based on where people live under the assumption that most strokes occur at home [31]. As previously detailed, our methods do not account for geographic boundaries such as rivers, mountains, weather, or traffic [32]. Finally, our model did not account for the capacity of each PSC center or the burden of acute stroke in an area. Unfortunately, the burden of acute stroke is complex, consisting of incident cases and acute cases of stroke recurrence. Current surveillance systems do not allow for regional estimates of the burden of acute stroke for all adults living in the US. Given that the rise of noncommunicable disease is likely to become the most pressing global health issue, one of World Health Organization's Action Plan objectives is to monitor noncommunicable diseases and their determinants and evaluate progress at the national, regional, and global levels [33].

In summary, we found that as age increased, the odds of having access to acute stroke care decreased. This was true for duplicate access, single access, any access, and potential access. Further research is needed to explore the role TM is playing in access to acute stroke care on a national level and ways to expand access to acute stroke care to provide coverage to one of groups at the highest risk for acute stroke, the elderly.

## Figures and Tables

**Table 1:** Current and potential access to stroke care in Texas by age category.

	Total population (*N* = 17,185,928)	Age <65 (*N* = 14,784,076)	Age ≥65 (*N* = 2,401,852)	*p* value
Access to care				<0.0001
Duplicate access	8,152,125	7,197,436 (48.7%)	954,689 (39.7%)	
Single access	3,330,146	2,792,829 (18.9%)	537,317 (22.4%)	
Potential access	2,875,892	2,511,632 (17.0%)	364,260 (15.2%)	
No access	2,827,765	2,282,179 (15.4%)	545,586 (22.7%)	

**Table 2:** Sixty-minute access to acute stroke care (stand-alone PSC or TM) and logistic model results for age in years (continuous variable).

	Coefficient	Std. error	*Z*	*p* value	Odds ratio (95% CI)
Model 1: age, no adjustment	−0.031	0.002	−16.09	<0.001	0.97 (0.97–0.97)
Model 2: adjusted for race, ethnicity, and sex	−0.011	0.002	−5.17	<0.001	0.98 (0.99–0.99)
Model 3: adjusted for race, ethnicity, sex, SES proxies, and insurance coverage	−0.016	0.002	−6.98	<0.001	0.98 (0.98–0.99)
Model 4: adjusted for race, ethnicity, sex, SES proxies, insurance coverage, urbanization, and total population	−0.007	0.003	−2.59	<0.001	0.99 (0.99–0.99)

**Table 3:** Multinomial model results for age in years (as a continuous variable) using no access to acute stroke care as referent.

	Coefficient	Std. error	*Z*	*p* value	Odds ratio (95% CI)
Model 1					
Duplicate access	−0.047	0.002	−19.05	<0.001	0.95 (0.95–0.96)
Single access	−0.049	0.003	−16.95	<0.001	0.95 (0.95–0.96)
Potential access	−0.039	0.003	−12.72	<0.001	0.96 (0.96–0.97)

Model 2					
Duplicate access	−0.032	0.003	−11.46	<0.001	0.97 (0.96–0.97)
Single access	−0.033	0.003	−10.21	<0.001	0.97 (0.96–0.97)
Potential access	−0.049	0.004	−13.92	<0.001	0.95 (0.95–0.96)

Model 3					
Duplicate access	−0.037	0.003	−12.11	<0.001	0.96 (0.96–0.97)
Single access	−0.029	0.003	−9.00	<0.001	0.97 (0.97–0.97)
Potential access	−0.042	0.004	−11.20	<0.001	0.96 (0.96–0.96)

Model 4					
Duplicate access	−0.027	0.005	−5.08	<0.001	0.97 (0.97–0.98)
Single access	−0.023	0.004	−6.54	<0.001	0.98 (0.97–0.98)
Potential access	−0.033	0.004	−7.54	<0.001	0.97 (0.96–0.97)

Model 1: age, no adjustment.

Model 2: adjusted for race, ethnicity, and sex.

Model 3: adjusted for race, ethnicity, sex, and SES proxies.

Model 4: adjusted for race, ethnicity, sex, SES proxies, urbanization, and total population.
